# Performance and characterization of the FinEstBeAMS beamline at the MAX IV Laboratory

**DOI:** 10.1107/S1600577521006032

**Published:** 2021-07-08

**Authors:** Kirill Chernenko, Antti Kivimäki, Rainer Pärna, Weimin Wang, Rami Sankari, Mats Leandersson, Hamed Tarawneh, Vladimir Pankratov, Mati Kook, Edwin Kukk, Liis Reisberg, Samuli Urpelainen, Tanel Käämbre, Frank Siewert, Grzegorz Gwalt, Andrey Sokolov, Stephanie Lemke, Svyatoslav Alimov, Jeniffa Knedel, Oliver Kutz, Tino Seliger, Mika Valden, Mika Hirsimäki, Marco Kirm, Marko Huttula

**Affiliations:** a MAX IV Laboratory, Lund University, PO Box 118, SE-22100 Lund, Sweden; b Nano and Molecular Systems Research Unit, University of Oulu, PO Box 3000, FI-90014 Oulu, Finland; c Institute of Physics, University of Tartu, W. Ostwald Street 1, EE-51014 Tartu, Estonia; d Computational Physics Laboratory, Tampere University, PO Box 692, FI-33014 Tampere, Finland; e Institute of Solid State Physics, University of Latvia, 8 Kengaraga iela, LV-1063 Riga, Latvia; f Department of Physics and Astronomy, University of Turku, FI-20014 Turku, Finland; g BESSY-II, Helmholtz-Zentrum Berlin für Materialien und Energie, Albert-Einstein-Straße 15, 12489 Berlin, Germany; h Surface Science Group, Laboratory of Photonics, Physics Unit, Tampere University, PO Box 692, FI-33014 Tampere, Finland

**Keywords:** MAX IV, photon energy resolution, photon flux, plane grating monochromator, beam polarization

## Abstract

FinEstBeAMS (Finnish–Estonian Beamline for Atmospheric and Materials Sciences) is a multidisciplinary beamline constructed at the 1.5 GeV storage ring of the MAX IV synchrotron facility in Lund, Sweden. The beamline covers an extremely wide photon energy range, 4.5–1300 eV, by utilizing a single elliptically polarizing undulator as a radiation source and a single grazing-incidence plane grating monochromator to disperse the radiation. At photon energies below 70 eV the beamline operation relies on the use of optical and thin-film filters to remove higher-order components from the monochromated radiation. This paper discusses the performance of the beamline, examining such characteristics as the quality of the gratings, photon energy calibration, photon energy resolution, available photon flux, polarization quality and focal spot size.

## Abstract 2

The performance of the new Finnish–Estonian Beamline for Atmospheric and Materials Sciences is characterized and discussed.

## 1. Introduction   

The FinEstBeAMS beamline at the MAX IV Laboratory was designed to fulfil the various needs of the Estonian, Finnish and other Nordic user communities in gas-phase electron and ion spectroscopies, surface science, and photoluminescence research, while also providing a modern platform to any users of synchrotron radiation with their own experimental end stations. Therefore, the new beamline had to afford an extensive photon energy range from ultraviolet to soft X-rays, good performance in terms of photon energy resolution and photon flux (often needed in gas-phase electron spectroscopy), reasonably small focal spot sizes to allow moderate spatial resolution in surface studies, and a possibility of probing samples with light of variable polarization. A further restriction in the design was to have only one undulator and one monochromator despite the extended operation range. A solution for how these demanding goals could be achieved was presented in the design paper of the FinEstBeAMS beamline (Pärna *et al.*, 2017 ▸).

The photon source of FinEstBeAMS is an elliptically polarizing undulator (EPU). It has a very long magnetic period, 95.2 mm, for the effective production of photons in the ultraviolet spectral region at the 1.5 GeV storage ring. The long magnetic period inevitably compromises the performance of the beamline in the soft X-ray region but usable flux (>10^10^ photons per second) is available up to 1000 eV with a good resolving power (*ca* 5000). The plane grating monochromator of the FinEstBeAMS beamline is of the SX700 type (Petersen & Baumgärtel, 1980 ▸). At present, two plane Au-coated gratings are available. The first one, with 600 lines mm^−1^ line density, can be used at photon energies from 15 to 1300 eV. The second plane grating operates in the photon energy range from 4.5 to 50 eV and has an exceptionally sparse line density of 92 lines mm^−1^. The combination of the low-line-density grating and grazing-incidence optics on the beamline results in poor high-order suppression in monochromated light. Therefore, each of the two beamline branches contains specific filter units, where six different filters can be mounted, purifying the radiation further for low-photon-energy experiments.

At present, FinEstBeAMS has three experimental end stations: the gas-phase end station (GPES) for electron and ion spectroscopy and for photoelectron–photoion coincidence spectroscopy of low-density matter (Kooser *et al.*, 2020 ▸), the solid-state end station (SSES) for photoelectron and X-ray absorption spectroscopy of surfaces and interfaces, and the photoluminescence endstation (PLES) for luminescence spectroscopy of solids (Pankratov *et al.*, 2019 ▸). As the vacuum requirements are significantly different in surface and gas-phase studies, the SSES and GPES have been placed on separate branch lines, which are accordingly called the solid-state and gas-phase branches. The PLES has been designed to be a movable end station and, when in use, it is installed on the gas-phase branch (in which case the GPES is moved upstream and the photon beam passes through it). The GPES and PLES have been in regular user operation since April 2019. The SSES accepts its first regular users in 2021.

In this paper, we give a short overview of the beamline design, report the results of the optical characterization of the plane gratings, and present the results of the beamline performance characterization, including photon energy resolution and photon flux in the accessible energy range, accuracy of the photon energy calibration, quality of the polarization properties of the photon beam, and focal beam spot size. We also discuss how the achieved performance meets the estimations made on the basis of optical element characterization.

## 2. Overview of the beamline   

**Figure 1:**
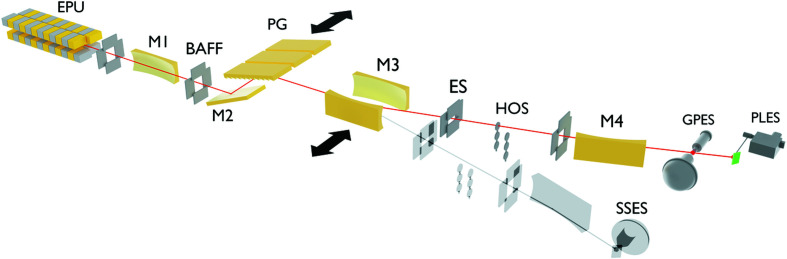
Schematic layout of the beamline optical system. The following abbreviations are used: EPU – elliptically polarizing undulator, M1–M4 – mirrors, BAFF – baffles, PG – plane grating, ES – exit slit, HOS – higher-order suppressing filters, GPES – gas-phase end station, PLES – photoluminescence end station, and SSES – solid-state end station.

The optical layout of FinEstBeAMS is shown in Fig. 1 ▸. The photon source, an elliptically polarized undulator (EPU) of the APPLE II type (Sasaki *et al.*, 1993 ▸), was built in-house (Thiel *et al.*, 2018 ▸; Tarawneh *et al.*, 2019 ▸) based on the design used at Helmholtz-Zentrum Berlin (HZB) (Bahrdt *et al.*, 2001 ▸). It contains four arrays of magnets that can be shifted independently in the longitudinal direction in order to control the radiation polarization. The beamline design follows the concept of the collimated plane grating monochromator (Follath, 2001 ▸). A side-cooled toroidal mirror (M1) is the first optical element in the beamline, mounted 12.00 m from the centre of the undulator. It collimates the beam in both directions, horizontally and vertically. After M1, two pairs (vertical and horizontal) of precise baffles (BAFF) are used to limit the monochromator aperture (acceptance angle). The monochromator was manufactured by FMB Feinwerk und Messtechnik GmbH, Berlin. It contains an internally cooled plane mirror (M2) and three slots for side-cooled plane gratings. Currently, two Au-coated plane gratings are installed (PG1, 600 lines mm^−1^ and PG2, 92 lines mm^−1^). The selected grating disperses incoming radiation in the vertical plane and – with the adjusted position and incidence angle of the M2 mirror – guides the central energy of the outgoing radiation in a direction parallel to the incoming beam. The branch line used in the experiments is selected by inserting either of the two toroidal focusing mirrors, M3GP or M3SS, into the beam path. That mirror focuses the dispersed radiation at the exit slit, which is located 6.00 m downstream from the focusing mirror. After the exit slit, each branch line contains a higher-order suppression unit (HOS), where two optical filters (fused silica, MgF_2_) and four thin-film metal filters (In, Sn, Mg, Al) are mounted on two linear manipulators; *i.e.* each manipulator has a holder for three filters. These sets of filters allow the suppression of higher-order radiation at photon energies below 72 eV. An ellipsoidal mirror (M4GP or M4SS) refocuses the monochromated radiation to the end station in each branch. It deflects the beam sideways, keeping it in the same horizontal plane. The entrance and exit arms of the refocusing mirror have been chosen to be 15% longer in the gas-phase branch than in the solid-state branch, so that the end stations are physically not mounted exactly side by side.

The beamline utilizes a standard MAX IV control system based on IcePAP motion controllers (Sjöblom *et al.*, 2016 ▸) and *Tango* software packages with the *Sardana* framework (Coutinho *et al.*, 2011 ▸).

## 3. Optics characterization   

The mirrors and grating blanks were provided by various commercial vendors, whereas the gratings were manufactured at the grating laboratory of HZB (Siewert *et al.*, 2018 ▸) using a mechanical diamond ruling method. First, the grating blanks were covered with a ruling gold layer and the line pattern was applied by a mechanical ruling process. The obtained pattern was then etched by an ion beam, forming a blazed surface. In the last step, the surface was coated with a reflective gold layer, which provides optimal efficiency in the required photon energy range. The design goal of covering the whole photon energy range with a single monochromator demands an extremely sparsely ruled grating for low photon energies. The optimal blaze angle for low-energy operation can be several degrees, which, combined with a low line density, results in a very large material deformation applied by the grating ruling machine. The ruling gold layer for the 92 lines mm^−1^ grating had to be about three times thicker than for the 600 lines mm^−1^ grating in order to allow ruling of a blaze angle of approximately 8°. The profile height of the ruled lines was increased from 225 nm for the 600 lines mm^−1^ grating to >1500 nm for the 92 lines mm^−1^ grating, resulting in more trenching during pattern transfer into silicon. This leads to rounding of a large part of the blaze facet, and identifying the final blaze angle by linear assumption does not result in the real grating profile.

Optical components installed in the beamline have to withstand the powerful beam generated by the EPU at FinEstBeAMS. Their quality is essential to ensure all the required beam characteristics, like a small focal spot size, polarization, spectral resolution and a sufficiently high flux of photons in a wide energy range. Therefore, it was decided to inspect the beam- and wave-front shaping parameters of all the mirrors and gratings to verify their performance. The micro-roughness on the surface of optical elements is of additional interest because it causes scattering and consequently a loss of photons. These studies were performed *ex situ* at the at-wavelength metrology facility at BESSY-II (Schäfers *et al.*, 2016 ▸) of HZB. The slope errors, and shape parameters like the radii of curvature, were studied by means of the BESSY-NOM (Siewert *et al.*, 2004 ▸). The NOM allows measurement of the slope error of reflective surfaces for the spatial frequency range from about 1 mm up to a full aperture length (Siewert *et al.*, 2016 ▸), which corresponds to a range of spatial frequencies sufficient to cause a deformation of the wavefront and thus have a direct impact on the coherent part of the photons. The mid- and high-spatial-frequency shape error was measured by means of a white-light interferometer (Wyant, 2002 ▸) using magnifications 10×, 20× and 50×. The micro-roughness on the grooves of the gratings was inspected by means of an atomic force microscope (Nanosurf Nanite). The parameters of the mirrors were presented in the design paper by Pärna *et al.* (2017 ▸). Table 1 ▸ shows the same parameters of the gratings as studied at the BESSY-II Optics Lab. One can see that the micro-roughness on the blaze facet is much higher for the 92 lines mm^−1^ grating. This is caused by the high ruling-layer thickness needed for the low-line-density grating: the gold grain roughness is transferred into the silicon. Usually, the gold of the blaze facets is compressed during the ruling process and a facet roughness of 0.8–1 nm r.m.s. for blaze angles ≥1.5° can be achieved. The gold grains in the 92 lines mm^−1^ profiles are only compressed in the surface region, leading to a much larger facet roughness.

**Figure 2:**
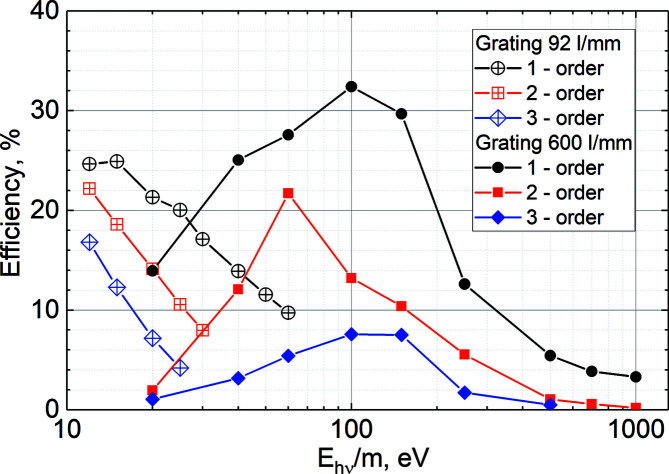
Efficiency of the 92 lines mm^−1^ (hollow points) and 600 lines mm^−1^ (filled points) gratings at the first (dots), second (squares) and third (diamonds) diffraction orders *m* measured using *c*_ff_ = 2.0.

The performance of both gratings was also characterized at the at-wavelength metrology facility at BESSY-II (Schäfers *et al.*, 2016 ▸) and using a reflectometer (Sokolov *et al.*, 2016 ▸). Fig. 2 ▸ shows the measured efficiencies of the two gratings in the energy range 20–1000 eV. The efficiencies for the second and third orders are given in ‘transmission’ mode, where a photon energy value is divided by the corresponding diffraction order *m*.

## 4. Beamline performance   

### 4.1. Photon energy repeatability and calibration   

The advantage of illuminating the grating with collimated light is that the grating angles α and β can be chosen freely. The ratio cosα/cosβ is called a fixed-focus constant (*c*_ff_) and it actually defines many of the properties of the dispersed radiation (Petersen, 1982 ▸; Petersen *et al.*, 1995 ▸). The primary operation mode of the monochromator uses *c*_ff_ = 2.25, which provides a good compromise between high-order suppression, photon energy resolution and photon flux. The monochromator performance presented below was obtained with *c*_ff_ = 2.25 unless otherwise mentioned. The control system, however, allows the operation of the monochromator with different *c*_ff_ values, thus giving the possibility of increasing the photon resolution or photon flux, if required by the user.

The angular range of the grating cradle limits the low-energy side of the 92 lines mm^−1^ grating to 4.4 eV and that of the 600 lines mm^−1^ grating to about 15 eV. The highest achievable energies are effectively determined by the acceptance of the M2 mirror: the practical limits in the primary operation mode are about 50 and 1300 eV for the 92 and 600 lines mm^−1^ gratings, respectively.

**Figure 3:**
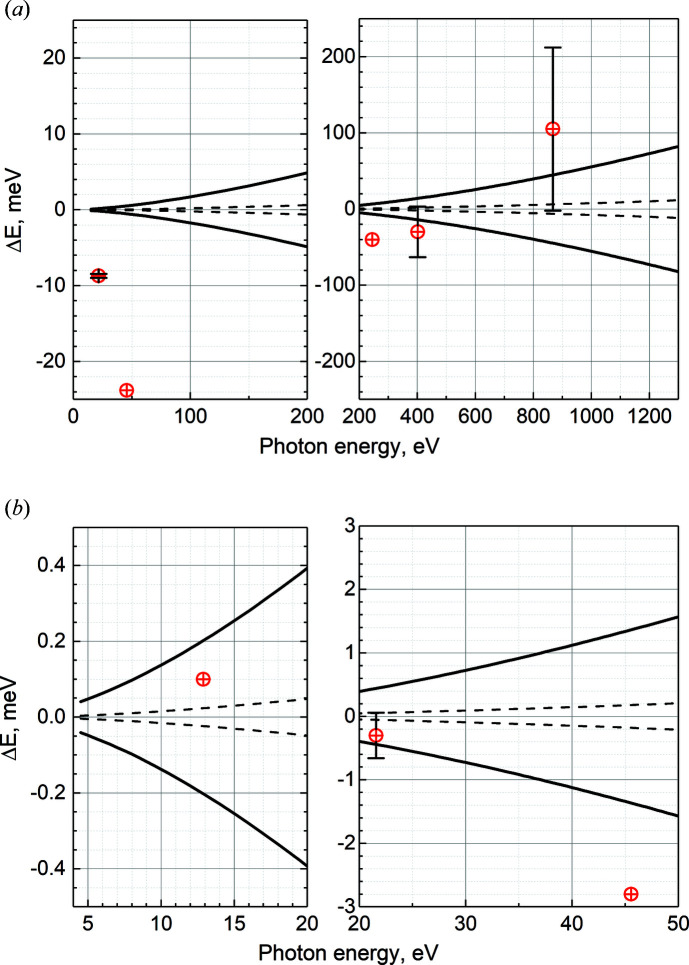
Photon energy repeatability limits and monochromator calibration accuracy for (*a*) the 600 lines mm^−1^ grating and (*b*) the 92 lines mm^−1^ grating. Dashed lines represent the photon energy repeatability calculated using the angular repeatability of the mechanical system. Solid lines show the positioning accuracy determined by the motion controllers. Red dots show a residual shift in photon energy after the calibration of the monochromator. The error bars of the red dots represent one standard deviation of the peak position that was obtained using different *c*_ff_ values.

According to the manufacturer’s data, the angular repeatability of the mechanical system is 0.24 µrad for the M2 mirror and 0.036 µrad for the gratings, which determines a limit for the photon energy repeatability. Fig. 3 ▸ shows the limits of the photon energy repeatability calculated for both gratings using the above-mentioned values of angular repeatability (dashed lines). During a measurement, the photon energy repeatability is also determined by the settings of the motion controllers, which are programmed to have a positioning tolerance (a dead band) to avoid high-frequency noise from the encoders and to have a reasonably short settling time. The dead band is 0.625 µrad for the motion controllers of both M2 and PG. The solid lines in Fig. 3 ▸ show the calculated photon energy repeatability, as determined by the settings of the motion controllers. As can be seen, errors in photon energy arising from the mechanical repeatability of the system, shown by dashed curves in Fig. 3 ▸, are practically negligible compared with errors arising from positioning repeatability defined by the motion controllers. Therefore, the solid lines in Fig. 3 ▸ provide information on a minimum step size to be used in a scan measurement. To obtain a reasonably even-spaced photon energy scale, the step size should not be less than the value of the solid line at the corresponding photon energy in the figure.

The photon energy scale has been calibrated by the method described by Weiss *et al.* (2001 ▸). The general idea is to measure the position of a gas absorption feature with a known energy using different *c*_ff_ values. For a perfectly aligned monochromator, the photon energy *E* diffracted to the exit slit is described by the grating equation,
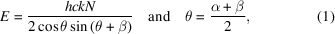
where *h* is Planck’s constant, *c* is the speed of light, *k* is the diffraction order, *N* is the line density of the grating, θ is the plane mirror angle, α is the incident angle and β is the diffraction angle. If the directional angles of M2 and PG differ from their ideal values, the photon energy observed at the exit slit, *E*_obs_, would not correspond to the expected value:
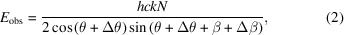
where Δθ and Δβ are the errors in the plane mirror and diffraction angles, respectively. Using equation (1), equation (2) can be expressed as a function of the photon energy, angular errors and *c*_ff_:
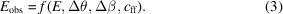
Therefore, if the energy *E* of the absorption feature is known, the experimentally obtained dependence of the observed peak position on the *c*_ff_ value can be approximated with equation (3), thus calculating the values of Δθ and Δβ. Then the errors in the directional angles of M2 and PG can easily be found.

The calibration method was implemented by measuring total ion yield (TIY) spectra with an ion time-of-flight (TOF) spectrometer installed at the GPES. The measurements were made using the N 1*s* → π*, *v* = 1 transition of N_2_ at 401.10 eV (Sodhi & Brion, 1984 ▸) for the 600 lines mm^−1^ grating and the 2*p*_1/2_ → 13*d* transition of Ne at 21.5806 eV (Ito *et al.*, 1988 ▸) for the 92 lines mm^−1^ grating. Several iterations of the calibration procedure were made until no systematic shift in the peak position was observed with the change in *c*_ff_. A residual shift that had no dependence on *c*_ff_ still remained between the observed and known photon energies. It arose from the misalignment of the roll angle of the M3 mirror and was corrected by changing that angle. After the calibration procedure, the following transitions were investigated with *c*_ff_ = 2.25: Ne 1*s* → 3*p* at 867.12 eV (Sodhi & Brion, 1984 ▸), Ne 2*p*_1/2_ → 13*d* at 21.5806 eV (Ito *et al.*, 1988 ▸), Ne 2*s* → 3*p* at 45.5442 eV (Schulz *et al.*, 1996 ▸), N_2_ N 1*s* → π*, *v* = 1 at 401.10 eV (Sodhi & Brion, 1984 ▸), Ar 2*p*_3/2_ → 4*s* at 244.39 eV (King *et al.*, 1977 ▸) and Xe 5*p*_1/2_ → 9*s* at 12.8887 eV (Berkowitz, 1979 ▸; Johnson *et al.*, 1980 ▸). The obtained residual shifts between the known and observed photon energies are represented in Fig. 3 ▸ by red dots. Some of these transitions were also re-measured with different *c*_ff_ values. Slight shifts in the observed peak positions were observed; however, the behaviour of the shifts was not systematic, but rather noise-like. As these energy shifts were reproducible, a conclusion was made that they arise because of the limit of absolute accuracy of the mechanical system, which is typically worse than its mechanical repeatability, thus representing an inherent characteristic of the monochromator. The error bars in black at chosen points in Fig. 3 ▸ represent one standard deviation of the peak positions obtained using different *c*_ff_ values.

Calibration can be considered excellent if error bars cross the zero level, which would mean that the photon energy scale is calibrated on the level of the mechanical accuracy of the monochromator. As can be seen from Fig. 3 ▸, this condition is not achieved in the low photon energy range of the 600 lines mm^−1^ grating. We believe that this residual shift arises from a slight vertical misalignment of the exit slit of the monochromator. Even in that case, features in both absorption spectroscopy and photoemission spectroscopy will show up very close to their expected energy positions. In high-accuracy experiments, a separate calibration of the photon energy scale is needed at the time of the measurement, no matter how well the general calibration of the monochromator is performed.

### 4.2. Photon energy resolution   

**Figure 4:**
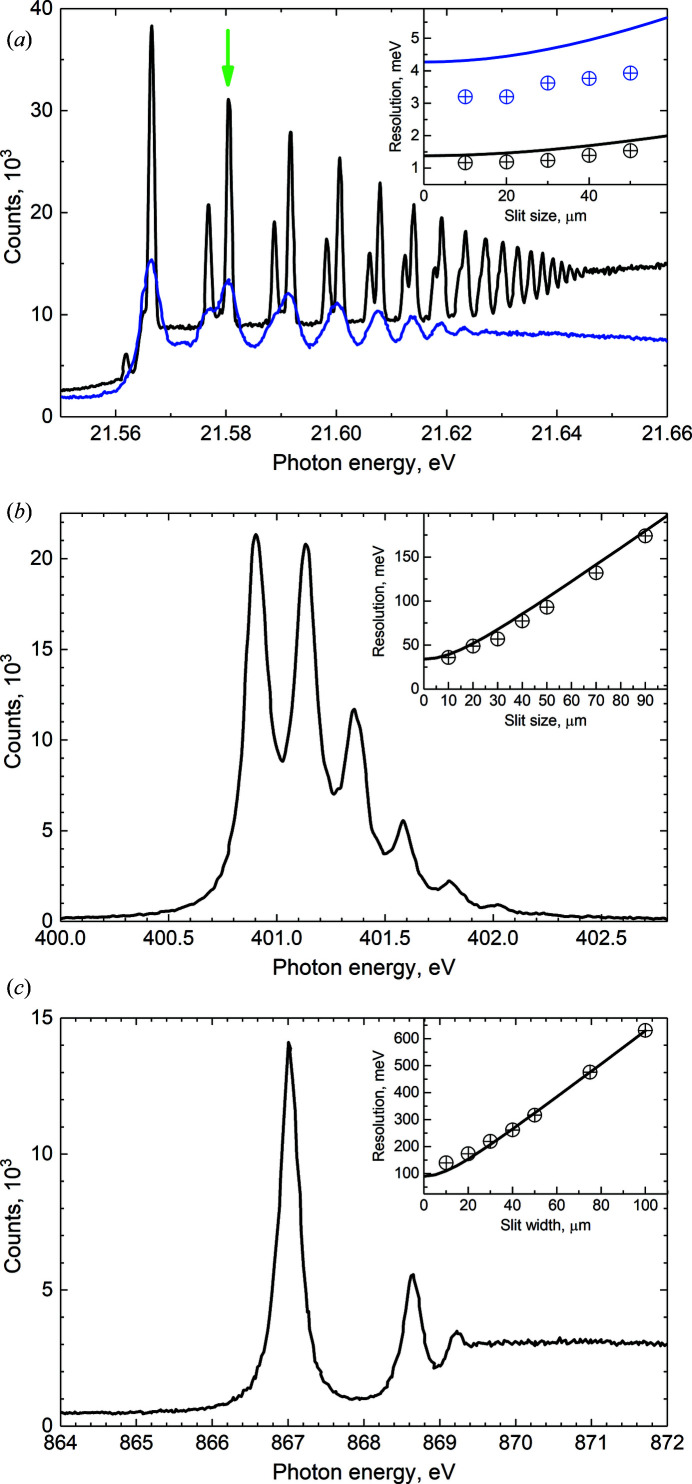
Total ion yield spectra of (*a*) Ne 2*p* → *nd,ms*, (*b*) N_2_ N 1*s* → π*, and (*c*) Ne 1*s* → *np* excitations measured using the 600 lines mm^−1^ grating (black line) and 92 lines mm^−1^ grating (blue line). (Insets) Experimentally obtained (dots) and calculated (solid lines) dependences of resolution on the slit width for the corresponding graphs.

The photon energy resolution of the beamline was investigated by measuring the TIY spectra of several gases at the GPES. For that purpose, the Ne 1*s* → 3*p*, Ne 2*p* → 13*d*, N_2_ N 1*s* → π*, Ar 2*p*_3/2_ → 4*s* and Xe 5*p*_1/2_ → 9*s* resonances were used. Some selected spectra are displayed in Fig. 4 ▸.

The experimental core-excitation spectra were fitted with Voigt profiles, where the Lorentzian widths in the final fits were fixed to the following values based on the data in the literature: 111 meV for the Ar 2*p*_3/2_ → 4*s* excitation (Kato *et al.*, 2007 ▸; Nicolas & Miron, 2012 ▸), 112 meV for the N 1*s* → π* excitations in N_2_ (Kato *et al.*, 2007 ▸) and 256 meV for the Ne 1*s* → 3*p* excitation (Coreno *et al.*, 1999 ▸; Kato *et al.*, 2006 ▸). These values do not represent our suggestions for the lifetime broadenings of the core-excited states concerned, but the sole purpose of this analysis was to estimate the photon energy resolution. The Ne 2*p* and Xe 5*p* excitation spectra were approximated by Gaussian functions, as the contribution of lifetime widths in these spectra was assumed to be negligible. The experimental photon energy resolutions, obtained with a 10 µm slit, and the corresponding resolving powers are presented in Table 2 ▸. The dependences of the photon energy resolution as a function of the monochromator exit slit width were measured as well. They are shown with dots for the corresponding spectra in the insets of Fig. 4 ▸.

The photon energy resolution of the beamline was estimated by taking into account four main factors: source size, exit slit width, slope errors of the optical elements, and the diffraction limit. For the sake of simplicity, it was assumed that all of these factors follow a Gaussian distribution, and thus quadratic summing can be used to estimate the overall resolution. It should be noted, however, that the spatial distribution of the source is not Gaussian (Onuki & Elleaume, 2002 ▸) and the contribution of the exit slit would be described more adequately by a rectangular function. The calculated resolution agrees well with the experimental values, except for the cases where the diffraction limit of the grating (at low energy range) or the exit slit width gives a dominant term to the photon energy resolution. In these cases, the calculations tend to overestimate the resolution, *i.e.* underestimate the resolving power. In the case of the exit slit contribution, this overestimation was taken into account by introducing a multiplier of 0.8 into the exit slit term. Examples of comparisons between the calculated (solid line) and experimentally obtained resolution (dots) are presented in the insets of Fig. 4 ▸.

**Figure 5:**
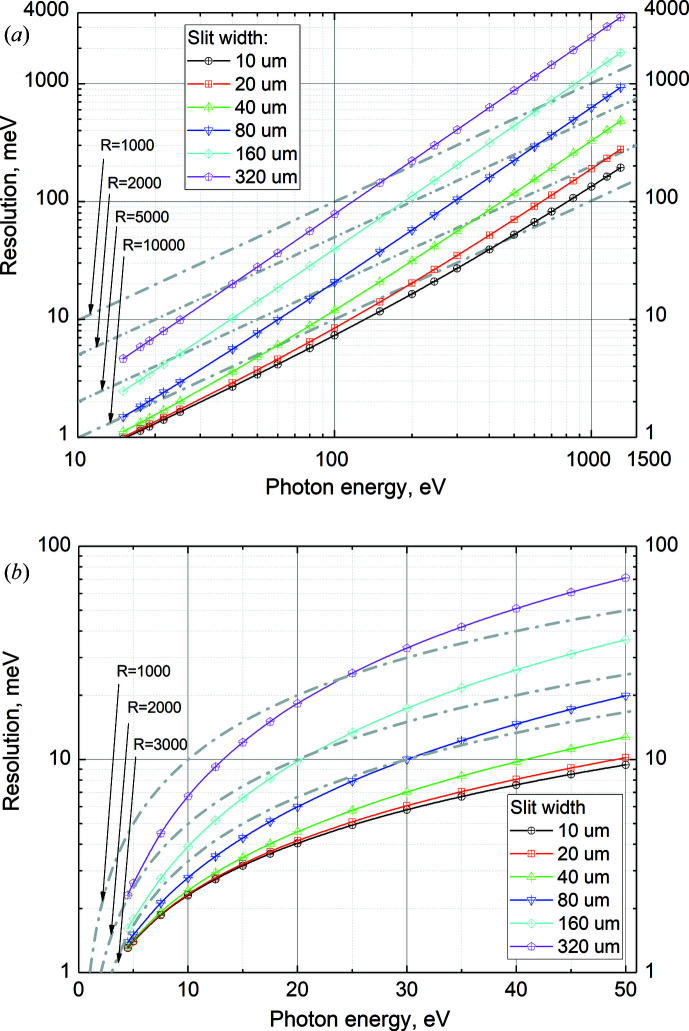
Calculated photon energy resolution for (*a*) the 600 lines mm^−1^ grating and (*b*) the 92 lines mm^−1^ grating. The corresponding values of slit widths are shown in the plot legends.

In this way, we could estimate the photon energy resolution over a wide energy range and with different values of slit widths (Fig. 5 ▸). The calculated values are expected to reproduce the experimental resolution well, apart from a slight overestimation in the low energy range. In Fig. 5 ▸, dashed–dotted lines are shown as eye guides for some values of constant resolving power.

### 4.3. Beam polarization   

The undulator contains four arrays of magnets that can be shifted independently in the longitudinal direction, allowing full control of the radiation polarization. So far, the undulator control system has been commissioned to operate in two modes, helical and inclined. In the inclined mode, two diagonal arrays of magnets move antiparallel, producing radiation with a zero phase shift between the horizontal and vertical components of the electric field vector, whereas in the helical mode the arrays of magnets move parallel, resulting in a phase shift of π/2. The magnitude of the magnets’ shift changes the ratio between the vertical and horizontal components of the electric field vector. In the inclined mode, it allows us to obtain linear horizontal and vertical polarizations, as well as to have linearly polarized light with a chosen angle of inclination. In the helical mode, it is possible to obtain circularly polarized light by equalizing both components of the electric field vector. Changing the direction of the magnets’ shift allows the generation of left or right circular polarization.

As the radiation propagates through the beamline, its polarization can change as the reflections at optical elements change the ratio between the vertical and horizontal components of the electric field vector and introduce an additional phase shift between them. Therefore, the polarization state of the radiation at the end of the beamline needs to be characterized and the undulator parameters adjusted, namely the magnetic gap and the longitudinal shift of the magnets, in order to counteract the changes introduced by the beamline optics. The exceptions are horizontal and vertical linear polarizations, as in these cases one of the components of the electric field vector equals zero, so the polarization state is not affected if all beamline components are properly aligned.

Polarization measurements in the UV range are often performed with a two-stage device, composed of a retarder stage and an analyzer stage with a coupled detector (Schledermann & Skibowski, 1971 ▸). The full characterization of polarization requires the acquisition of a two-dimensional intensity map with retarder and analyzer angles as independent variables, resulting in a significant number of individual measurements. We adopted a less time-consuming method where only a single analyzer mirror was used, similar to the method described by Cubric *et al.* (1999 ▸): if the radiation is fully polarized, this provides information about linear and circular polarization.

It can be shown that the dependence of the intensity of the polarized radiation *I* on the analyser mirror angle η is described by the equation
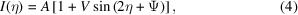
where the visibility *V* and phase shift Ψ are parameters defined by the radiation polarization and the properties of the mirror. Note that visibility is a function of photon energy.

If one assumes that light is fully polarized (*S*_0_ = 1), the Stokes parameters can be found as:
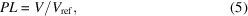


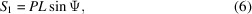


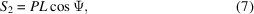


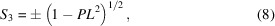
where *V*_ref_ is the reference visibility value, which is the visibility for fully linearly polarized radiation. The reference visibility value can be obtained from separate measurements using horizontal or vertical polarization as they are not affected by the beamline. The parameter *PL* in these equations is the linear polarization degree, 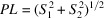, which characterizes the amount of linear polarization. The sign of *S*_3_ defines right or left polarization helicity. It cannot be found from the present measurement but can be easily predicted by calculations.

As mentioned before, the described method is only valid when the radiation is fully polarized, *S*_0_ = 1. Calculations made using measured magnetic fields in the undulator gap predict a high degree of polarization: the amount of unpolarized radiation is in the range of 0.1–0.7% for photon energies from 4.5 to 120 eV. To test the degree of polarization experimentally, the He 1*s* photoelectron spectrum was measured with horizontal and vertical linear polarizations at photon energies of 40 eV (with the 92 lines mm^−1^ grating) and 100 eV (with the 600 lines mm^−1^ grating). The Scienta R4000 electron analyzer of the GPES was positioned in the horizontal direction and it was operated with a pass energy of 10 eV. Analysis of the measured spectra intensities gave values of 99.46% (at 40 eV) and 99.17% (at 100 eV) for the degree of linear polarization. These values are underestimates of the real degree of polarization because the electron lens of the electron spectrometer has a large acceptance angle of 16°.

Utilizing the method described above, the undulator parameters were optimized in the range from 20 to 200 eV for two cases: circular polarization (both left and right), and inclined polarization with a ‘magic angle’ (54.7°) between the horizontal direction and electric field vector.

**Figure 6:**
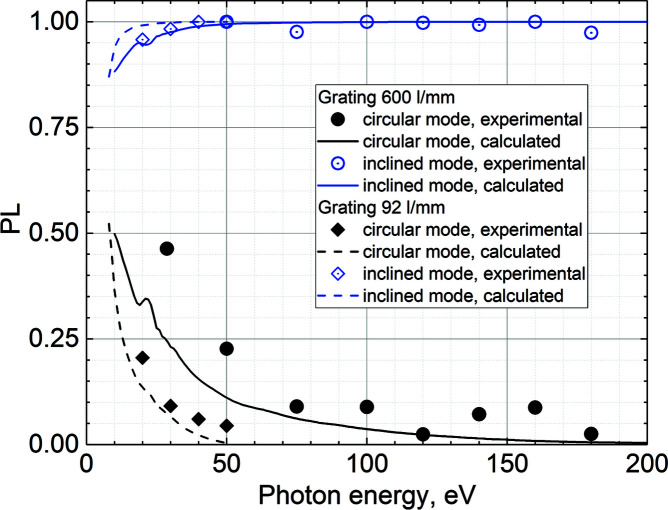
Linear polarization degree *PL* for circular polarization (black filled points) and inclined polarization (blue hollow points) measured with the 600 lines mm^−1^ (dots) and 92 lines mm^−1^ (diamonds) gratings. Solid and dashed lines of the corresponding colour show calculated dependences of *PL* for the 600 and 92 lines mm^−1^ gratings, respectively.

As the phase shift induced by the beamline cannot be reversed in the inclined and helical modes of the undulator, the resulting polarization state will, strictly speaking, always be elliptical. We can characterize the quality of the polarization state by its linear polarization degree *PL*. Fig. 6 ▸ shows the energy dependence of the degree of linear polarization measured (dots) and calculated (lines) for circular (black) and inclined (blue) polarizations. Circles and solid lines represent measurements and calculations for the 600 lines mm^−1^ grating, whereas diamonds and dashed lines represent measurements and calculations for the 92 lines mm^−1^ grating. Qualitatively, the experimental behaviour of *PL* is in good agreement with the calculations, even though the calculations seem to underestimate the phase delay introduced by the beamline optics. Using the 600 lines mm^−1^ grating, the polarization quality remains quite good down to 50 eV. At lower photon energies, the incident angles on M2 and the grating become steep, which introduces a large phase delay. The 92 lines mm^−1^ grating is operated at much shallower incident angles. Its use preserves an acceptable quality of the polarization at low photon energies, but at the cost of lower photon energy resolution.

The EPU of FinEstBeAMS will be upgraded to operate in a universal mode (Bahrdt & Wüstefeld, 2011 ▸), which will allow the phase shift of the undulator to be adjusted as well. After its implementation we expect to provide pure circular and inclined polarization of radiation over the full energy range.

**Figure 7:**
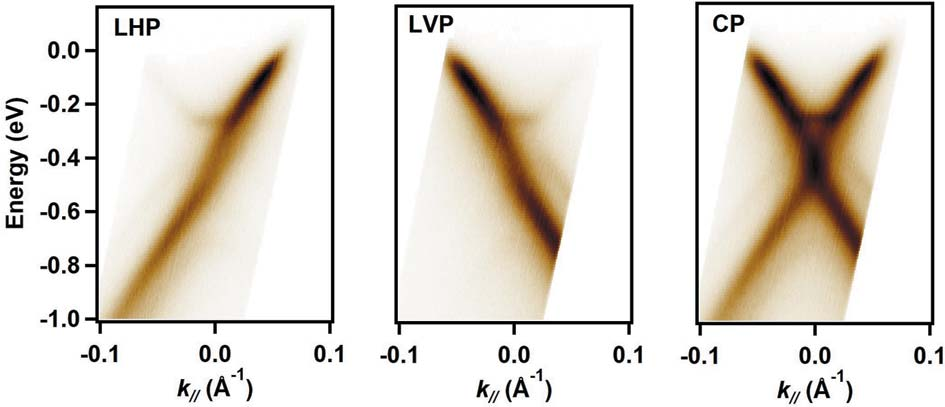
ARPES spectra of a mixed monolayer–bilayer graphene sample measured using radiation with the linear horizontal (LHP), linear vertical (LVP) and circular (CP) polarization. The data were taken along the 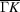 direction in the surface Brillouin zone of graphene using a photon energy of 22 eV. The 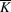 point is set as a reference 0 in momentum space (*k*_//_).

The possibility of using different polarizations greatly expands the experimental potential of the beamline. For example, the phenomenon of a ‘dark corridor’ in graphene is believed to be the result of a photoemission interference effect, verified in experiments using linearly polarized radiation (Bostwick *et al.*, 2007 ▸; Mucha-Kruczyński *et al.*, 2008 ▸); the electronic chirality near the von Hove singularity in graphene has been studied using circular polarized radiation (Hwang & Hwang, 2015 ▸; Liu *et al.*, 2011 ▸). Fig. 7 ▸ presents angle-resolved photoelectron spectroscopy (ARPES) data of a mixed monolayer–bilayer graphene on a 6H-SiC(0001) sample measured at the SSES. The obtained result agrees very well with the data found in the literature (Marchenko *et al.*, 2018 ▸), indicating a high degree of circular polarization at the end of the beamline.

### 4.4. Photon flux   

The undulator produces its maximum power (2.4 kW at the minimum gap of 14.0 mm) and provides the widest operational energy range in the horizontal polarization mode. Horizontal polarization is also considered to be the default operation mode for experiments.

Apart from the exit slit width, the photon flux that can be exploited in an experiment depends on a combination of the monochromator input acceptance and selected photon energy (also relative to the central cone energy of the used undulator harmonic). This combination also determines other important properties of the photon beam. For instance, the use of the central cone energy and a relatively small acceptance allows one to reach a small focal spot size in the experiments and reduces the contribution of even undulator harmonics. On the other hand, selecting a photon energy below the central cone energy and a larger acceptance allows one to achieve a higher photon flux.

In general, it is a complicated task to optimize the beamline parameters to obtain desirable beam properties among photon energy resolution, photon flux, size and shape of the beam spot, and presence of even harmonics, especially taking into account that different applications have rather diverse requirements for the photon beam. For example, photon flux and resolution are often the most crucial parameters in photoelectron spectroscopy, whereas the spot size and presence of higher-order radiation may not play important roles. In contrast, photoluminescence excitation measurements are significantly disturbed by the presence of higher-order radiation in the photon beam, whereas they do not really need the highest photon energy resolution provided by the monochromator because the studied luminescence spectra are influenced by solid-state broadening effects. Also, our experience indicates that high photon fluxes provided by an undulator often cannot be used due to excitation density effects (see Kirm *et al.*, 2009 ▸) and the limited radiation hardness of the samples.

**Figure 8:**
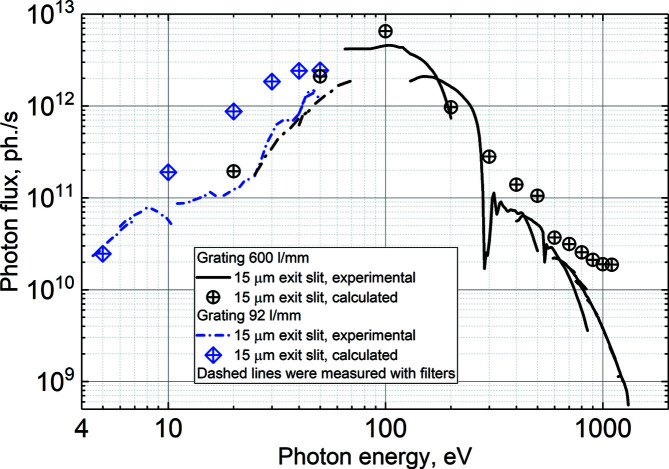
Photon flux at the end station with 300 mA ring current. Black and blue curves represent the photon flux obtained using the 600 and 92 lines mm^−1^ gratings, respectively, with a fixed 15 µm exit slit width. Dashed–dotted parts of the lines represent measurements done with filters. Circles and diamonds show the photon flux calculated for a constant exit slit of 15 µm using the 600 and 92 lines mm^−1^ gratings, respectively.

As a reference, we present photon flux curves measured with an acceptance of 215 µrad × 260 µrad (horizontal × vertical) in the photon energy range 4.5–500 eV and with an acceptance of 90 µrad × 135 µrad in the photon energy range 500–1300 eV (Fig. 8 ▸) when the storage ring was operating with a 300 mA electron beam current.

The current response from an Opto Diode AXUV-100 photodiode installed in the PLES was used to obtain the value of the photon flux. This response was corrected for the diode’s quantum efficiency taken from the manufacturer’s data sheet. In the photon energy range 4.5–70 eV, different filters were used in order to reduce the impact of higher diffraction orders (curves obtained with the respective filters are represented with dashed–dotted lines in Fig. 8 ▸). For the same reason, the photon energy set by the monochromator was chosen to be higher than the peak energy value of the corresponding undulator harmonic, allowing us to decrease the intensity ratio between the second- and first-order radiation at low photon energies. For photon energies higher than 70 eV, the monochromator was tuned to the photon energy corresponding to the maximum of the undulator harmonic. In order to obtain the highest possible photon flux, successive undulator harmonics were used, namely the first, third, seventh and eleventh in the energy range 4.5–600 eV, and the 21st, 23rd, 25th, 27th, 31st, 35th, 39th and 43rd in the energy range 600–1300 eV.

In Fig. 8 ▸, black and blue curves represent the measured photon fluxes obtained with the 600 and 92 lines mm^−1^ gratings, respectively, using a fixed monochromator exit slit width of 15 µm. These data are compared against the simulated results (dots and diamonds). The undulator flux was estimated using the *SPECTRA* software (Version 10.2; Tanaka & Kitamura, 2001 ▸) for the harmonics described earlier. The ray-tracing model in *RAY-UI* (Baumgärtel *et al.*, 2016 ▸, 2019 ▸) was utilized to estimate the transmission of a photon entering the acceptance aperture to the sample plane. This transmission, multiplied with the undulator flux obtained in *SPECTRA*, presents the simulated flux expected on the sample. Note that all the figure errors of the delivered optical components were included in the ray-tracing simulations, and the actual acceptance used in the measurements was implemented in the calculations of the source characteristics as well as in the ray tracing.

As can be seen in Fig. 8 ▸, the correspondence between the experimental and simulated results is better for the measurement taken with the 600 lines mm^−1^ grating. However, there is a discrepancy on the higher energy side, the difference being about an order of magnitude at 1000 eV. The differences between the experiment and the simulations can be attributed to contamination of the mirrors and gratings. A clear indication of carbon contamination is seen in the measured photon flux at around 280 eV. The calculated photon flux at low energies also does not take into account the transmission of the filters that were used for the measurements at energies below 50 eV. The transmission of the filters varies in a range approximately from 10 to 80% depending on the type of filter. To reduce the effect of carbon contamination, a steady oxygen flow will be applied to the mirrors receiving a significant load of the synchrotron light. Such a method, called oxygen cleaning, has been shown to be effective on other beamlines at MAX IV.

The photon flux increases proportionally with increasing exit slit width. Using Figs. 8 ▸ and 5 ▸, one can estimate the photon flux and resolution for a chosen value of the exit slit width and find the best compromise between them suitable for the required experiment.

### 4.5. Focal beam spot size   

Gas-phase electron spectroscopy and photoluminescence techniques do not have strict requirements for the beam spot size; they can be satisfied with a beam spot of a couple of hundred micrometres without significant losses in performance. In contrast, electron spectroscopy of surfaces and interfaces, conducted at the SSES, could benefit from a smaller beam spot. However, a high beam divergence in the low energy range and space constraints in the experimental hall resulted in the choice of a relatively small demagnification factor of 2.6 for the M4 mirrors on both branches and modest spot sizes at the end stations. With an expected horizontal spot size of 220–320 µm at the monochromator exit slit, the focal beam spot size should be of the order of 100 × 100 µm in the major part of the operation range for a beam unrestricted horizontally and monochromator exit slit sizes of several hundred micrometres. A pair of horizontal baffles has been installed before the monochromator exit slit, allowing us to decrease the horizontal size of the beam spot if needed in some applications.

**Figure 9:**
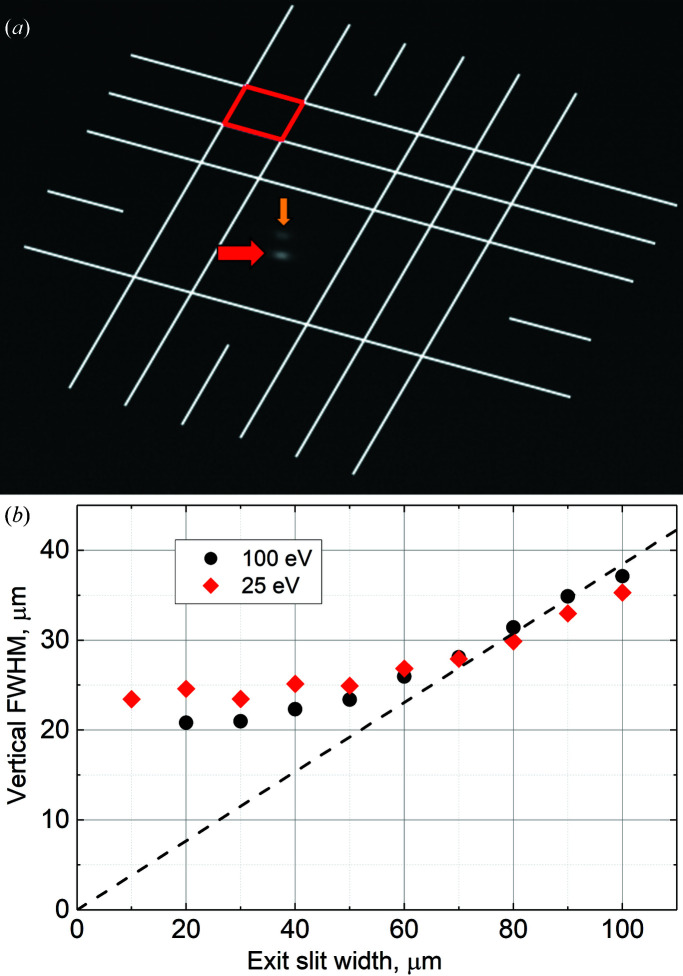
(*a*) An image of a focal beam spot on a scaled YAG:Ce crystal with an exit slit width of 100 µm × 100 µm. The red quadrilateral unit represents a 200 µm^2^ square on the YAG:Ce crystal. The red and yellow arrows point to the focal beam spot on the front and back surfaces of the crystal, respectively. (*b*) The dependence of the vertical beam spot size on the monochromator exit slit width measured at photon energies of 25 eV (diamonds) and 100 eV (dots). An ‘ideal’ dependence (exit slit width divided by a demagnification coefficient) is shown by the dashed line.

For the beam spot visualization, an yttrium aluminium oxide garnet crystal doped with cerium (YAG:Ce) was installed on a sample holder in the analysis chamber of the SSES. The crystal had a metal grid on the surface with 1 mm step and 200 µm ticks for size reference. A long-focus optical microscope with a CCD camera was mounted on a viewport of the analysis chamber to record images. Photographs were taken through the viewport at an angle relative to the measurement plane, which resulted in quadrilateral scale units [Fig. 9 ▸(*a*)]. White lines were added to the photographs in order to scale separately the line profiles of the horizontal and vertical spot sizes. The beam spot size was then determined as the full width at half-maximum (FWHM) of the beam spot profile.

Fig. 9 ▸(*b*) presents the dependence of the vertical beam spot size on the monochromator exit slit width at photon energies of 25 and 100 eV. As can be seen, the vertical beam size deviates from the ideal dependence for slit widths below 80 µm and approaches a constant value of 20–25 µm. The horizontal beam spot size of the unrestricted beam practically shows no dependence on photon energy and remains in the range of 80–100 µm for photon energies above 10 eV. With the use of the horizontal baffles at the exit slit, it was possible to limit the horizontal beam spot size down to about 30 µm.

These values are in good agreement with the ray-tracing calculations presented by Pärna *et al.* (2017 ▸), where a beam spot of 100 µm × 20 µm was predicted using a 50 µm monochromator exit slit width and a horizontally unrestricted beam at a photon energy of 17 eV.

## 5. Conclusion   

The baseline commissioning of the FinEstBeAMS beamline has been completed. The monochromator of the beamline is equipped with a 92 lines mm^−1^ grating for the photon energy range 4.5–50 eV and a 600 lines mm^−1^ grating for the photon energy range 15–1300 eV. A resolving power of ∼5000 has been achieved at 13 eV photon energy with the 92 lines mm^−1^ grating and of ∼11000 at 400 eV photon energy with the 600 lines mm^−1^ grating. Overall, the photon energy resolution agrees well with calculations based on the parameters of the optical elements. For the convenience of users, we have presented in graphical form the estimated photon energy resolution for different slit widths over the whole operation range of the FinEstBeAMS beamline.

Four polarization modes are available for users: horizontal linear and vertical linear polarizations, circular polarization (left and right), and ‘magic angle’ inclined linear polarization, mostly intended for gas-phase users. Using horizontal polarization, the beamline can deliver above 2 × 10^11^ photons s^−1^ in the photon energy range below 10 eV at a resolving power of 3000, and more than 10^13^ photons s^−1^ at a resolving power of 5000 in the photon energy range 50–150 eV. At higher photon energies, the photon flux decreases, but it is still ∼10^9^ photons s^−1^ at 1253.6 eV. A spot size of ∼90 µm (horizontal) × 25 µm (vertical) has been measured at the solid-state end station, in good agreement with design values.

FinEstBeAMS has accepted regular users at the gas-phase and photoluminescence end stations since April 2019. Regular user operation at the SSES begins in 2021. Future commissioning work of the beamline will focus on circular and inclined polarization modes, optimization of the beamline acceptance, oxygen cleaning of the optics and characterization of beamline performance using different fixed-focus constants.

## Figures and Tables

**Table 1:** Parameters of the plane gratings

Optical element	PG1	PG2
Shape	Plane	Plane
Surface size (L × W, mm)	140 × 25	140 × 25
Substrate	Si	Si
Coating (thickness, nm)	Au (32)	Au (31)
Tangential radius *R* (km)	72	61
Sagittal radius *r* (km)	≥20	≥20
Slope error (µrad r.m.s.)	0.19	0.21
Micro-roughness σ (nm r.m.s.)	0.17–0.33	≤0.2
Micro-roughness on the blaze facet σ_g_ (nm r.m.s.)	0.1	7.9 ± 1.5
Groove density (lines mm^−1^)	600	92
Blaze angle (°)	1.90	4.2

**Table 2:** Photon energy resolution and corresponding resolving power values obtained using a 10 µm exit slit width

		92 lines mm^−1^ grating		600 lines mm^−1^ grating	
Transition	Energy (eV)	Resolution (meV)	Resolving power	Resolution (meV)	Resolving power
Xe 5*p*_1/2_→9*s*	12.89	2.58	5000		
Ne 2*p*_1/2_→13*d*	21.59	3.20	6700	1.17	18400
Ar 2*p*_3/2_→4*s*	244.39			28	8600
N_2_ N1*s*→π*	401.10			36	11000
Ne 1*s*→3*p*	867.12			140	6200
